# Simultaneously Improving Ductility and Stretch Formability of Mg-3Y Sheet via High Temperature Cross-Rolling and Subsequent Short-Term Annealing

**DOI:** 10.3390/ma15134712

**Published:** 2022-07-05

**Authors:** Yinyang Wang, Chen Liu, Yu Fu, Yongdong Xu, Zhiwen Shao, Xiaohu Chen, Xiurong Zhu

**Affiliations:** Ningbo Branch of Chinese Academy of Ordnance Science, Ningbo 315103, China; wangyinyang929@126.com (Y.W.); fuyuayu@126.com (Y.F.); lourry@163.com (Z.S.); xiaohuzss@126.com (X.C.); zxr0922@163.com (X.Z.)

**Keywords:** microstructure, texture, mechanical properties, stretch formability, high temperature cross-rolling, annealing

## Abstract

In this work, Mg-3Y sheet was prepared by high temperature cross-rolling and subsequent short-term annealing. The effect of annealing on microstructure, texture, mechanical properties, and stretch formability of Mg-3Y sheet was primarily investigated. Micro-nano size coexistence of β-Mg_24_Y_5_ phases can be well deformed with matrix. The as-rolled Mg-3Y sheet exhibited a homogeneous deformation microstructure consisting of deformed grains with extensive kink bands and dispersed β-Mg_24_Y_5_ phases. A double peak texture character appeared in as-rolled Mg-3Y sheet with a split of the texture peaks of about ±20° tilted to rolling direction. After annealing, the as-annealed Mg-3Y sheet presented complete static recrystallized (SRXed) microstructure consisting of uniform equiaxed grains. The texture orientation distribution was more dispersed and a weakened multiple-peak texture orientation distribution appeared. In addition, the maximum intensity of basal plane decreased from 5.2 to 3.1. The change of texture character was attributed to static recrystallization (SRX) induced by kink bands and grain boundaries. The as-annealed Mg-3Y sheet with high Schmid factor (SF) for basal <a> slip, prismatic <a> slip, pyramidal <a> slip, and pyramidal <c+a> slip exhibited high ductility (~25.6%). Simultaneously, enhanced activity of basal <a> slip and randomized grain orientation played a significant role in decreasing anisotropy for the as-annealed Mg-3Y sheet, which contributed to the formation of high stretch formability (~6.2 mm) at room temperature.

## 1. Introduction

As the lightest structural material, current magnesium (Mg) alloy sheets cannot meet the requirements of automotive vehicles due to poor room temperature formability [1]. The poor formability and limited ductility of Mg alloys at ambient temperature is ascribed to their hexagonal closed-packed crystal structure and the associated insufficient independent slip systems [2,3,4,5]. Under hot deformation conditions, more slip systems would be operated in addition to the basal slip system and new fine grains could be concurrently developed [3,4,5,6]. These can lead to a pronounced improvement in the ductility and plastic workability of Mg products at not only high temperatures, but also warm and cold temperatures. However, strong basal texture would develop in rolled Mg alloy sheets. The basal planes are mainly parallel to the RD-TD plane, where the RD and TD are rolling and transverse directions [7,8,9]. The basal slip and extension twinning during deformation play a dominant role in the development of basal texture. These two mechanisms align the c-axis of grains with the direction of compressive strain, i.e., in as-rolled sheets, c-axis parallel with the normal direction (ND). The strong basal texture is difficult to remove during subsequent annealing [10]. Therefore, it is necessary to find a way of weakening the texture in wrought Mg alloys.

One of the methods of texture control is the addition of rare-earth (RE) metals in the Mg alloys [11,12,13,14], which could modify and weaken the intensity of the basal texture of rolled Mg alloys. Another approach is the improvement of the plastic processing techniques. In recent years, some processing techniques, such as asymmetric rolling [15,16], cross-rolling [17], repeated unidirectional bending [18], repetitive bending [19], equal channel angular rolling [20], wavy roll forming [21], high temperature annealing before and after warm rolling [22], combination of high temperature and warm rolling [23,24], have been explored for texture control of Mg alloy sheets. Compared with those processing techniques, high temperature rolling exhibits different deformation characteristics, such as enhanced activities of non-basal slips and grain boundary sliding (GBS), which could effectively weaken the texture [25,26]. It was found that high temperature rolling and subsequent annealing can significantly improve the stretch formability [27,28] and deep drawability [29] of Mg alloys at room temperature. Some reports have also shown that basal texture of Mg alloys could be suppressed by cross-rolling [30,31,32]. Meanwhile, a decrease in anisotropy in cross-rolling sheet was reported [33,34]. 

As mentioned above, Mg alloys with RE elements subjected to high temperature rolling and cross-rolling have potential to achieve high ductility and high stretch formability at room temperature. Therefore, in the present study, the Mg alloys with Y addition are processed by combination of high temperature rolling and cross-rolling under different conditions (hot rolling condition and hot rolling-annealing condition). The pure Mg is served as the control sample. The microstructure, texture, mechanical properties, and stretch formability of Mg-Y alloys are investigated in detail.

## 2. Materials and Methods

**Figure 1:**
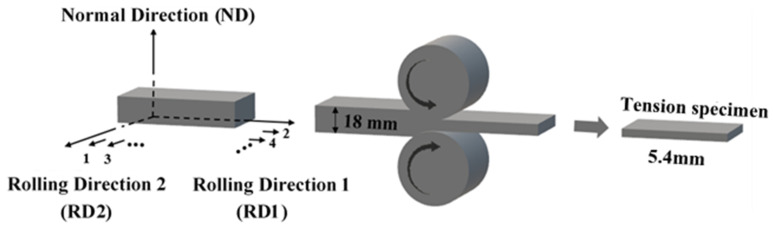
The schematic diagrams of hot cross-rolling.

**Figure 2:**
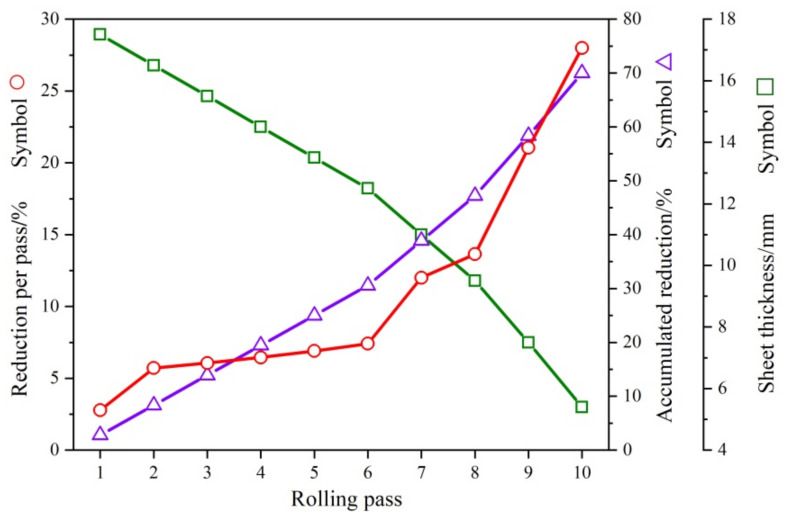
The reduction per pass, accumulated reduction, and sheet thickness as a function of rolling pass (red circle, blue triangle and green box represent reduction per pass, accumulated reduction and sheet thickness respectively).

Commercially pure Mg and Mg-3Y (wt%) master alloys were used to prepare the pure Mg and Mg-3Y (wt%) alloys. The fusion metallurgy was carried out in a mild steel crucible placed in an electric resistance furnace under an anti-oxidizing flux. After melting, the melt was cast into a steel mold at 993 K. The as-cast alloys were homogenized at 723 K for 24 h, then cooled down in the air. The alloys were machined into a rectangular shape with dimensions of 100 mm × 80 mm × 18 mm. Before hot rolling, the as-homogenized samples were preheated at 773 K for 30 min. Each rolling direction of cross-rolling changed at 90°, as shown in Figure 1. To reduce strain hardening during the rolling, an incremental duration per pass was adopted, as shown in Figure 2. The rolling samples were inter-pass annealing treated at the rolling temperature for 10 min after each pass. After a total reduction of 70%, the as-rolled sheets were cooled in the air. The recrystallization annealing treatment was conducted at 748 K for 15 min for the final as-rolled alloy sheet.

The chemical compositions of alloys obtained by an X-ray fluorescence analyzer (XRF, XRF-1800) are listed in Table 1. The phase analysis and macro-texture test were performed by an X-ray diffraction (XRD, Empyrean) with Cu Kα radiation. The microstructure was observed by an optical microscope (OM, Leica MEF4) and a scanning electron microscope (SEM, SUPRA 55) equipped with energy dispersive X-ray spectroscopy (EDS). Electron backscattered diffraction (EBSD) analyses were carried out using a field emission scanning electron microscope (SEM, ZEISS EV55) equipped with an HKL EBSD detector. Substructure analysis was examined by transmission electron microscope (TEM, Tecnai G220 S-Twin). The specimens for the OM and SEM observation were prepared following a standard procedure of grinding, polishing, and etching (1.0 g picric acid+2 mL acetic acid+3 mL water+20 mL ethanol).

**Figure 3:**
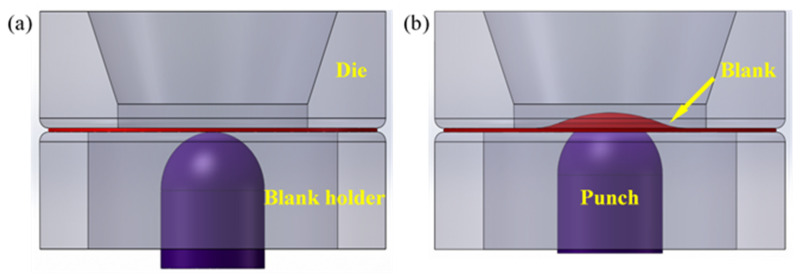
Schematic diagrams of the Erichsen test setup and punch position are shown (**a**) before and (**b**) after the test.

Tensile test was carried out using a DNS100 universal testing machine with a strain rate of 1 × 10^−3^ s^−1^ at room temperature. For each material, three parallel samples with their long axes parallel to the RD2 were tested. The fracture surface was also observed by the SUPRA 55 SEM. Erichsen test was carried out to determine the stretch formability of all parallel samples in each test using GBW-60Z Erichsen testing machine with a punch speed of 1 mm/min at room temperature. Schematic diagrams of the Erichsen test were shown in Figure 3. Samples for Erichsen test were machined into dimensions of 60 mm in length, 60 mm in width, and 1 mm in thickness.

## 3. Results and Discussion

### 3.1. Microstructure of the As-Cast Alloy

**Figure 4:**
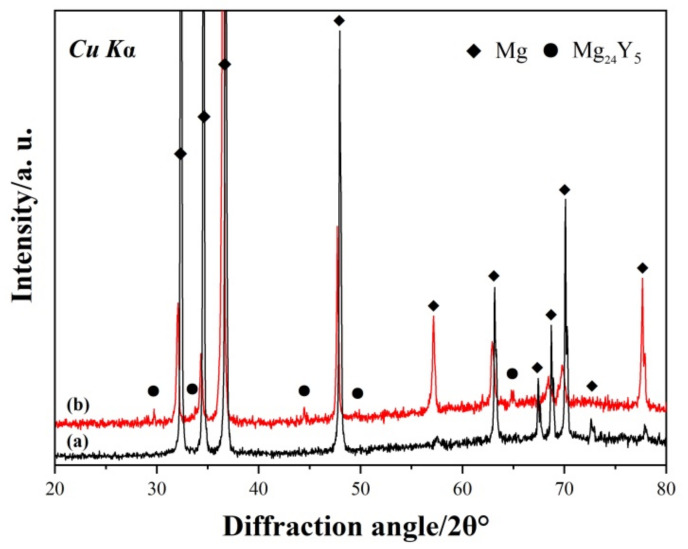
XRD patterns of the as-cast (a) pure Mg and (b) Mg-3Y alloys.

The XRD patterns of the as-cast pure Mg and Mg-3Y alloys are shown in Figure 4. Both samples contain primary α-Mg phase. The additional diffraction peaks corresponding to the β-Mg_24_Y_5_ phases can be indexed in the Mg-3Y alloy.

**Figure 5:**
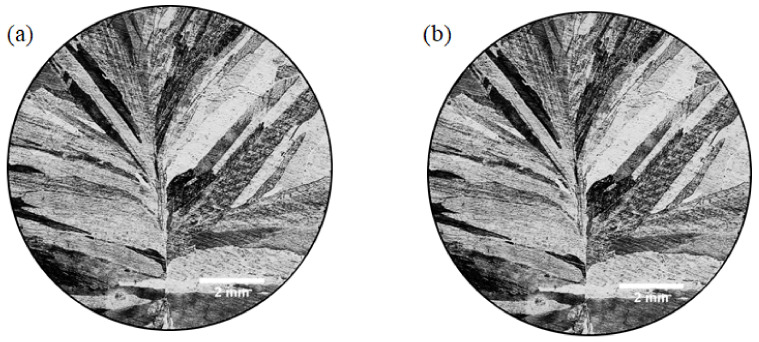
Macrostructures of the as-cast (**a**) pure Mg and (**b**) Mg-3Y alloys.

The macrostructures of as-cast pure Mg and Mg-3Y alloys are presented in Figure 5. The grain morphology changes from columnar grain to equiaxed grain and the grain size is clearly refined due to the addition of yttrium. The β-Mg_24_Y_5_ phases with a cubic crystal structure (lattice constant a = 11.257 Å) cannot act as the heterogeneous nuclei of α-Mg matrix [35]. However, the solute yttrium distributed on the solid–liquid interface could drag the dendrites growth during the growth of α-Mg grains, and thus refine the grains [36]. The growth restriction factor (GRF) could also be employed to describe the refining ability of solute elements in magnesium alloys. The GRF value of yttrium in magnesium is calculated to 1.7, which indicates that yttrium would have a certain refining ability [37].

**Figure 6:**
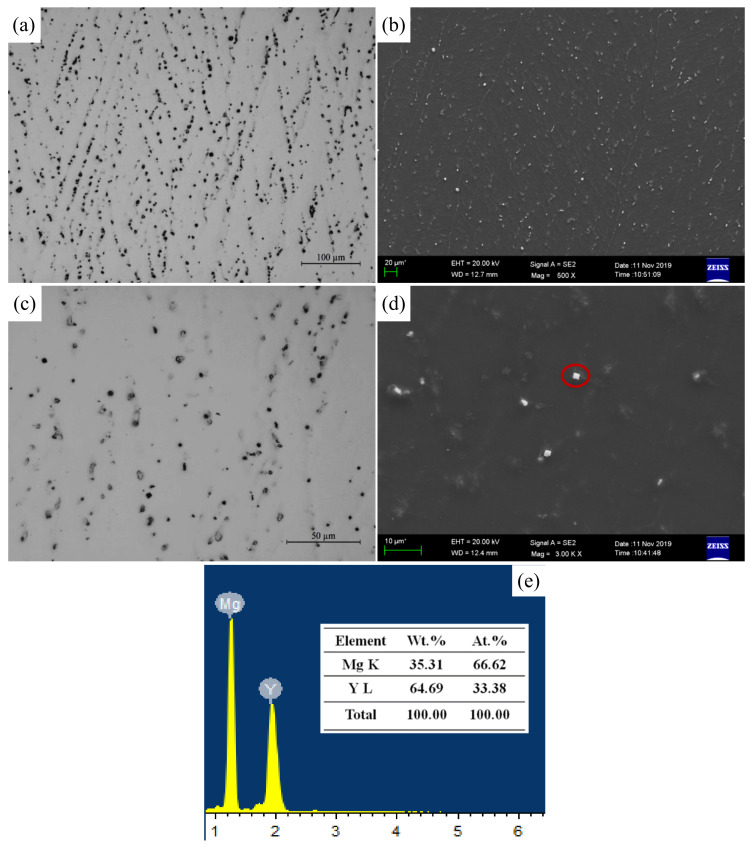
Optical micrographs of (**a**) low magnification view and (**b**) high magnification view; SEM micrographs of (**c**) low magnification view and (**d**) high magnification view; (**e**) the EDS results corresponded to the secondary phase marked by the red circle of as-cast Mg-3Y alloy.

The typical as-cast microstructure of the Mg-3Y alloy is shown in Figure 6. In Figure 6a,c, the Mg-3Y alloy is composed of α-Mg phase and numerous eutectic β-Mg_24_Y_5_ phases, which are discretely distributed between the dendrite arms. Most of the β-Mg_24_Y_5_ phases are bulk-shaped phases with different sizes, as shown in Figure 6b,d. The EDS result further confirms that the second phases existing in Mg-3Y alloy are β-Mg_24_Y_5_ phases, as shown in Figure 6d.

**Figure 7:**
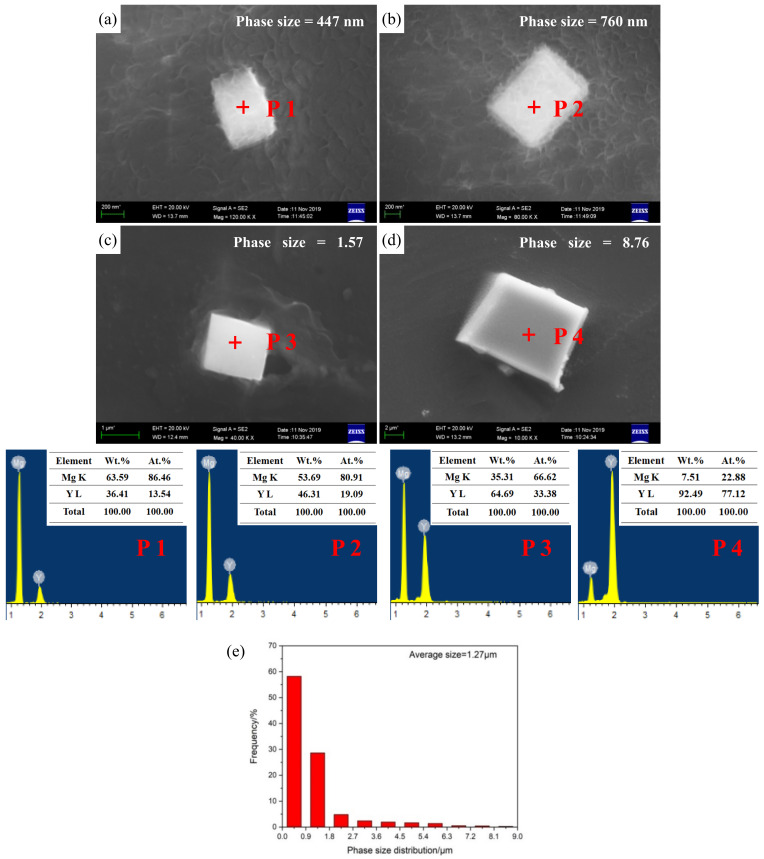
SEM micrographs and EDS results of the secondary phases with different sizes of (**a**,**b**) nano-scale, (**c**,**d**) micro-scale, and corresponding phase size distribution histograms (**e**) of as-cast Mg-3Y alloy.

The typical bulk-shaped β-Mg_24_Y_5_ phases with different sizes, from nanometer to micrometer, are presented in Figure 7. The size distribution of phases in nano-scale (Figure 7a,b) primarily varies from 225 to 675 nm (Figure 7e), accounting for about 58% of the total. In addition, the size distribution in micro-scale (Figure 7c,d) mainly changes from 1.12 to 2.48 μm (Figure 7e), accounting for about 34%. Although the size of the β-Mg_24_Y_5_ phases is fine, there is no clear aggregation. The morphological characteristic of β-Mg_24_Y_5_ phases with micro-nano size coexistence in matrix is formed finally.

**Figure 8:**
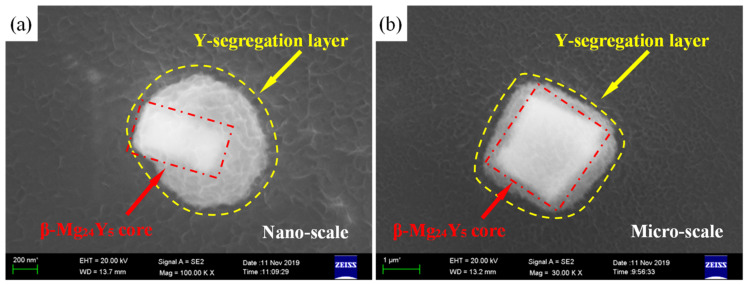
SEM micrographs of the secondary phases with Y-segregation layer of (**a**) nano-scale and (**b**) micro-scale of as-cast Mg-3Y alloy.

Apart from micro-nano size coexistence characteristic, the Y-segregation layer is also observed at both nano- and micro-scales, as shown in Figure 8a,b. The β-Mg_24_Y_5_ phase core is surrounded by the semi-transparent light grey layer. It is speculated that the semi-transparent light grey layer might be an Y-segregation layer, which could provide further growth and development of β-Mg_24_Y_5_ phases. When the concentration of Y-segregation layer reaches a certain value, the β-Mg_24_Y_5_ phase core would consume the Y-segregation layer to further grow and develop. The β-Mg_24_Y_5_ phases with relatively larger size (about ≥2 μm) have no clear Y-segregation layer in Figure 7, which indicates the well development of β-Mg_24_Y_5_ phases. Therefore, the micro-nano size coexistence of β-Mg_24_Y_5_ phases should be relevant to the solidification segregation behavior of Y [38]. The segregation of Y during the solidification process results in the formation of Y-rich region (micro-phase) and Y-depleted region (nano-phase).

**Figure 9:**
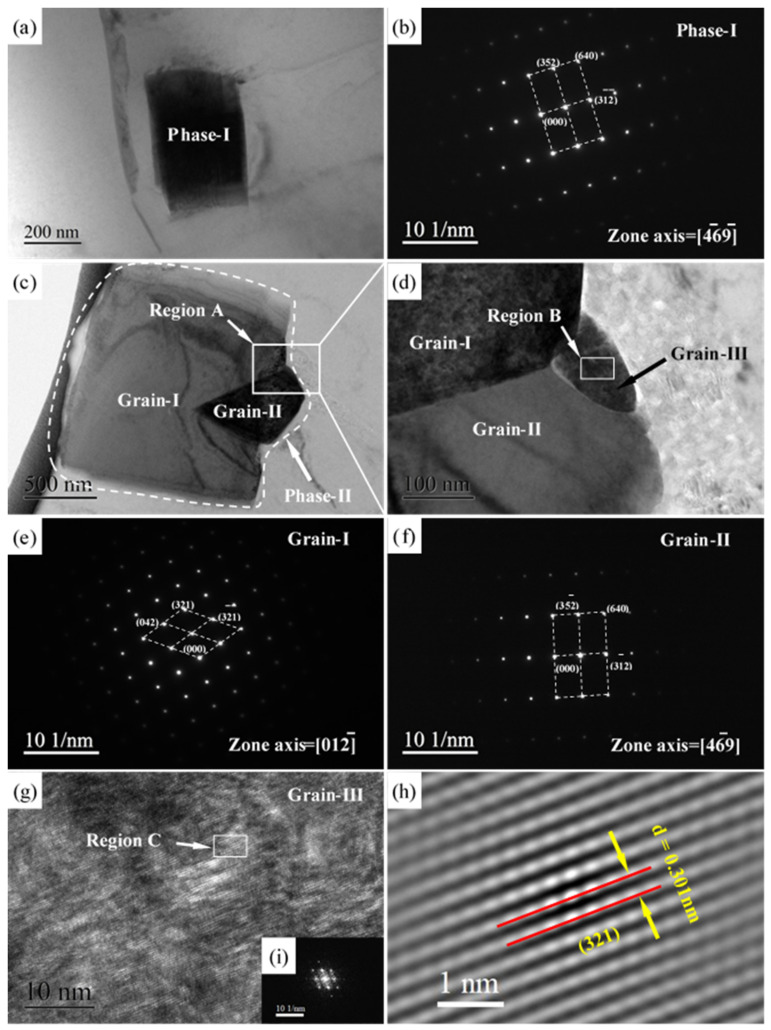
Bright field TEM micrographs of (**a**) phase-I, (**c**) phase-II, and (**d**) local magnification corresponding to region A; SAED patterns of (**b**) phase-I, (**e**) grain-I, and (**f**) grain-II; (**g**) HRTEM micrograph of region B in grain-III; (**i**) FFT pattern; and (**h**) inverse FFT micrograph of region C.

The nano- and micro-phase were further explored by TEM analysis. Figure 9a,b presents the bright-field TEM micrograph of nano phase-I and typical selected area electron diffraction (SAED) pattern of phase-I, respectively. It demonstrates that nano phase-I displays a rectangular shape, which is in good agreement with the morphology of β-Mg_24_Y_5_ phase, as shown in Figure 7a,b. In addition, the nano phase-I is a single crystal. The typical SAED pattern further ensures that phase-I corresponds to the β-Mg_24_Y_5_ phases. Notably, the interface between phase-I and Mg matrix is clear, which reveals that β-Mg_24_Y_5_ phases have good bonding with the matrix. Figure 9c,d shows the bright-field TEM micrograph of micro phase-I and local magnification of region A, respectively. Similarly, the micro phase-I also displays a rectangular shape and good bonding with the matrix. However, the micro phase-II consists of three parts (grain I–III) in addition to a single crystal. The β-Mg_24_Y_5_ phases are verified by the SAED pattern of grain-I and grain-II in Figure 9e,f. The high-resolution TEM (HR-TEM) observation was also performed at region B of grain-III, as can be seen in Figure 9g. The fast Fourier transform (FFT) and inverse FFT were conducted at the selected region C, correspondingly, as shown in Figure 9i,h. On the basis of the inverse FFT micrograph, the inter-planar spacing of grain-III is 0.301 nm, which is well consistent with the inter-planar spacing of ideal (321) plane for Mg_24_Y_5_ crystal. Consequently, the micro phase-II is polycrystalline with three grains rather than the clustering of nano-phases. The nano grain-III is covered by grain-I and grain-II. At the same time, the nano grain-II is covered by micro grain-I. This phenomenon may indicate that the micro-phase is developed from the nano-phase, which is well consistent with the solidification segregation behavior of Y, as mentioned above.

### 3.2. Microstructures and Micro-Texture Evolution of Alloy Sheets

**Figure 10:**
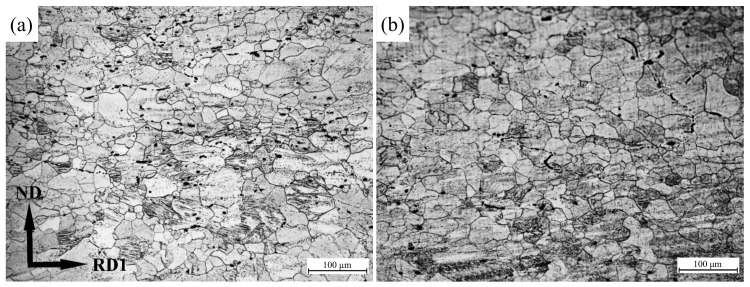
Optical micrographs of (**a**) as-rolled and (**b**) as-annealed pure Mg sheets.

The optical micrographs of Mg-R and Mg-RA sheets are shown in Figure 10. The microstructure of as-rolled samples is heterogeneous with a distribution in grain size from several μm to hundreds of μm. Apart from some dynamic recrystallized (DRXed) grains induced via grain boundary, many twins exist in coarse deformed grains. For as-annealed samples, the heterogeneity of grain size is faded along with grain coarsening.

**Figure 11:**
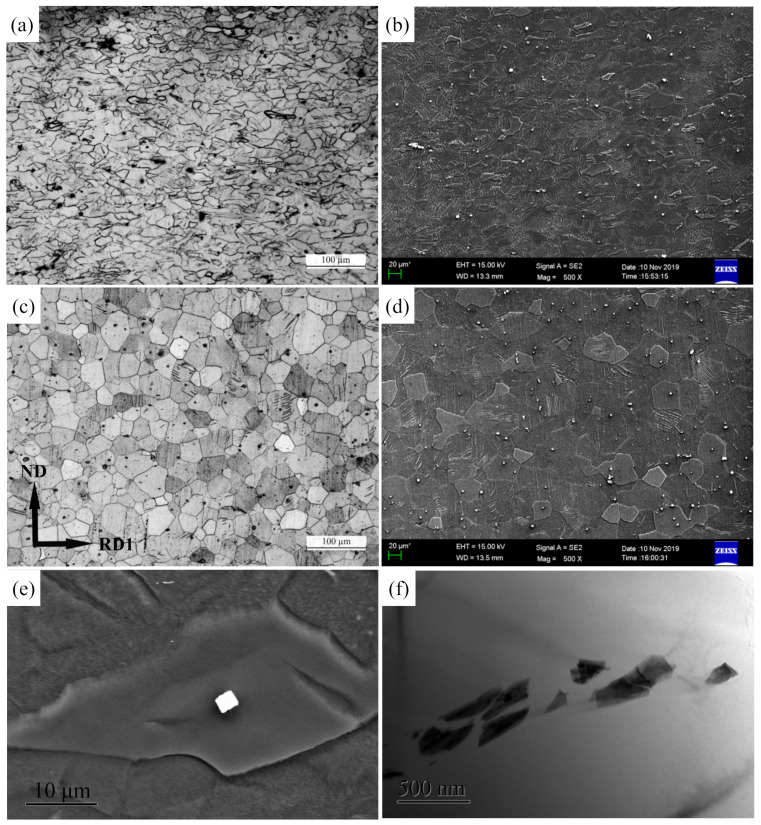
Optical micrographs of (**a**) as-rolled, (**c**) as-annealed, and corresponding SEM micrographs of (**b**) as-rolled and (**d**) as-annealed Mg-3Y sheets, the secondary phases; (**e**) SEM micrograph and (**f**) TEM micrograph of Mg-3Y sheets.

Figure 11a–d shows the microstructure of Mg-3Y-R and Mg-3Y-RA sheets. The microstructure of Mg-3Y-R sheet contains deformed grains dotted with fine and dispersed β-Mg_24_Y_5_ phases. The β-Mg_24_Y_5_ phases exist both at grain boundaries and in the interior of grains. Micron sized β-Mg_24_Y_5_ phases have no clear change and preserve the original morphological characteristic under as-cast condition (Figure 11e). Moreover, no apparent dynamic recrystallization (DRX) is found. In fact, the large second-phase particles (>1 μm) are the ideal sites for the development of DRX nuclei by particle stimulated nucleation (PSN) [39,40]. However, the PSN effect is absent around the micron-sized β-Mg_24_Y_5_ phases (~2.74 μm), as shown in Figure 11e. The fragmentation of β-Mg_24_Y_5_ phases is observed in Figure 11f, which indicates that nano-sized phases with weak bonding have been broken to some extent during rolling. The directional distribution of dispersed β-Mg_24_Y_5_ phases emerges. After annealing, the deformed grains change to equiaxed grains with uniform size for Mg-3Y-RA sheet due to static recrystallization (SRX). The distribution characteristic of β-Mg_24_Y_5_ phases has no significant difference from Mg-3Y-R sheets. In comparison with the Mg-R and Mg-RA sheets (see Figure 10), the Mg-3Y-R and Mg-3Y-RA sheets exhibit considerably more homogenous microstructures favoring further deformation.

**Figure 12:**
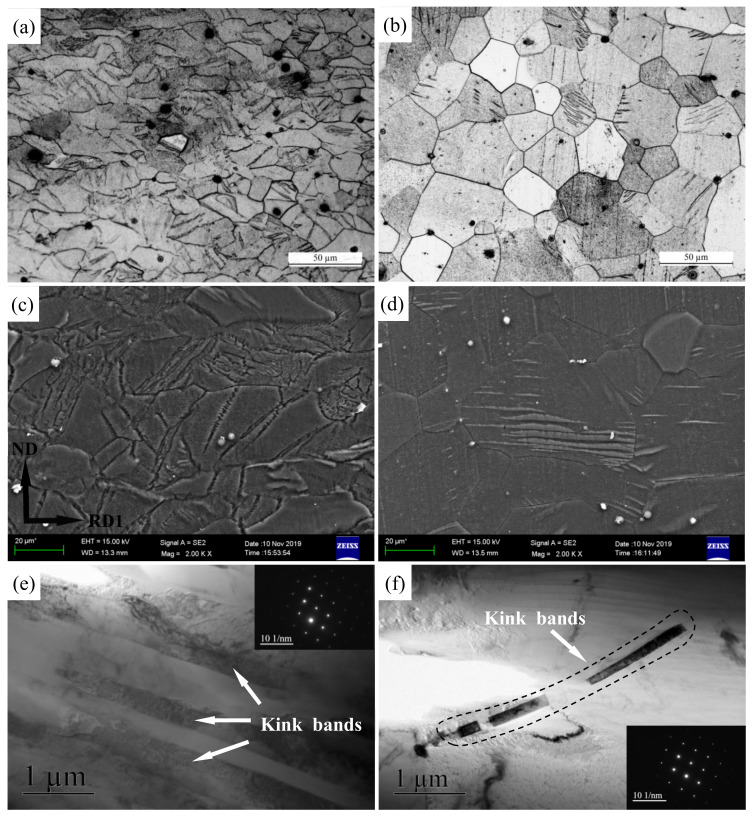
Optical micrographs of (**a**) as-rolled, (**b**) as-annealed, and corresponding SEM micrographs of (**c**) as-rolled and (**d**) as-annealed Mg-3Y sheets; TEM micrographs of kink bands of (**e**) as-rolled and (**f**) as-annealed Mg-3Y sheets.

The twinning is depressed with increasing deformation temperature [41] and extensive wrinkled structures occur in Mg-3Y-R sheet rolled at a quite high temperature of 773 K, as shown in Figure 12a,c. The wrinkled structures present lath structures with different contrasts in TEM. No diffraction spots for twins are detected and the interface of these lath structures is not as straight as twin boundaries, as indicated by Figure 12e. Therefore, these lath structures should be deformation bands, but twins. The deformation bands are almost parallel and the top is thin and sharp. Yang X Y et al. [5,42] believed that the deformation bands are kink bands, which correspond to a low angle dislocation interface. The kink bands are formed by bending the slip surface when the primary slip is blocked. The formation of kink bands is closely related to crystal orientation, deformation temperature, and deformation degree. Similar results have been reported by Zhou B et al. [43] and Matsumoto T et al. [44]. After annealing, the density number of kink bands decreases remarkably (Figure 12b,d) and the residual kink bands are discontinuous (Figure 12f).

**Figure 13:**
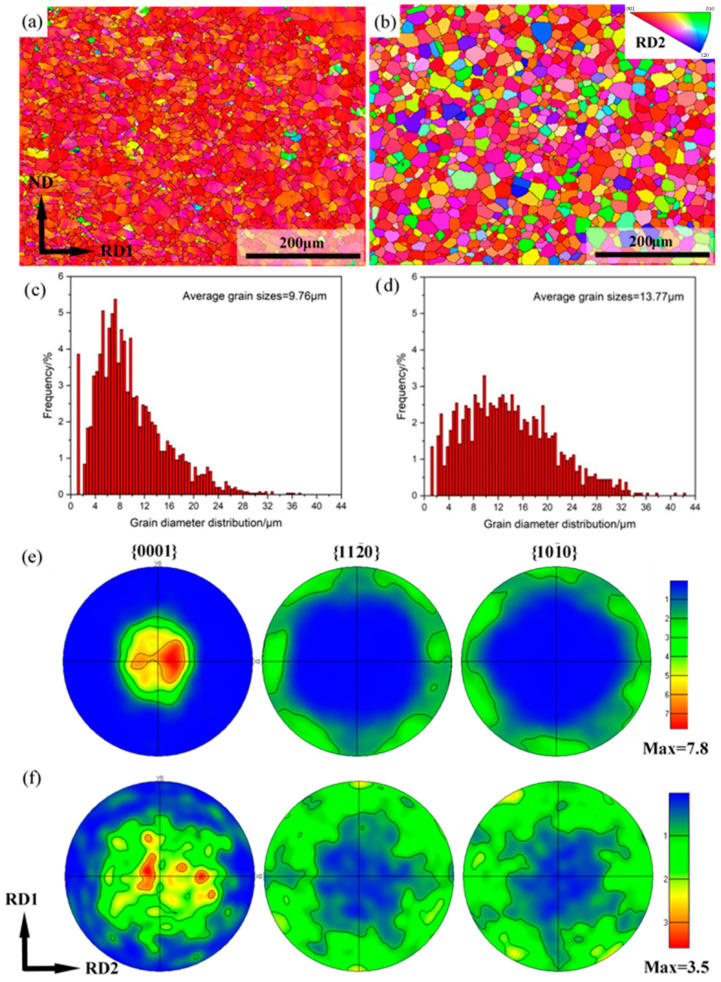
EBSD IPF maps of (**a**) as-rolled and (**b**) as-annealed; grain size distributions of (**c**) as-rolled and (**d**) as-annealed; (0001), (11-20), and (10-10) pole figures of (**e**) as-rolled and (**f**) as-annealed Mg-3Y sheets. Observation along RD2 was applied to IPF triangle.

EBSD measurements were conducted to further analyze the evolution of the microstructure and micro-texture on the RD1-ND plane of the alloy sheets. Figure 13a–d presents the EBSD inverse pole figure (IPF) maps and corresponding grain size distribution of Mg-3Y sheets. Different colors represent different orientations of the grains. The grains with similar color indicate that the misorientation angles between these grains are not significant in Mg-3Y-R sheets. In addition, the average grain size is about 9.76 μm. After annealing, the grains with multicolor intuitively show the weakening of the preferred orientation of grains. Moreover, the average grain size increases to about 13.8 μm. Figure 13e,f shows (0001), (11-20), and (10-10) pole figures of Mg-3Y sheets. The micro-texture characteristic of Mg-3Y-R sheet is the (0002) basal texture with a split of basal pole along the RD2 direction. The (0002) basal texture is weakened for Mg-3Y-RA sheet after annealing. First, the basal pole splits into multiple peaks and the orientation distribution becomes broader. The (11-20) and (10-10) planes have a random orientation distribution. Second, the maximum pole intensity decreases from 7.8 to 3.5 multiples of random orientation distribution (MRD).

**Figure 14:**
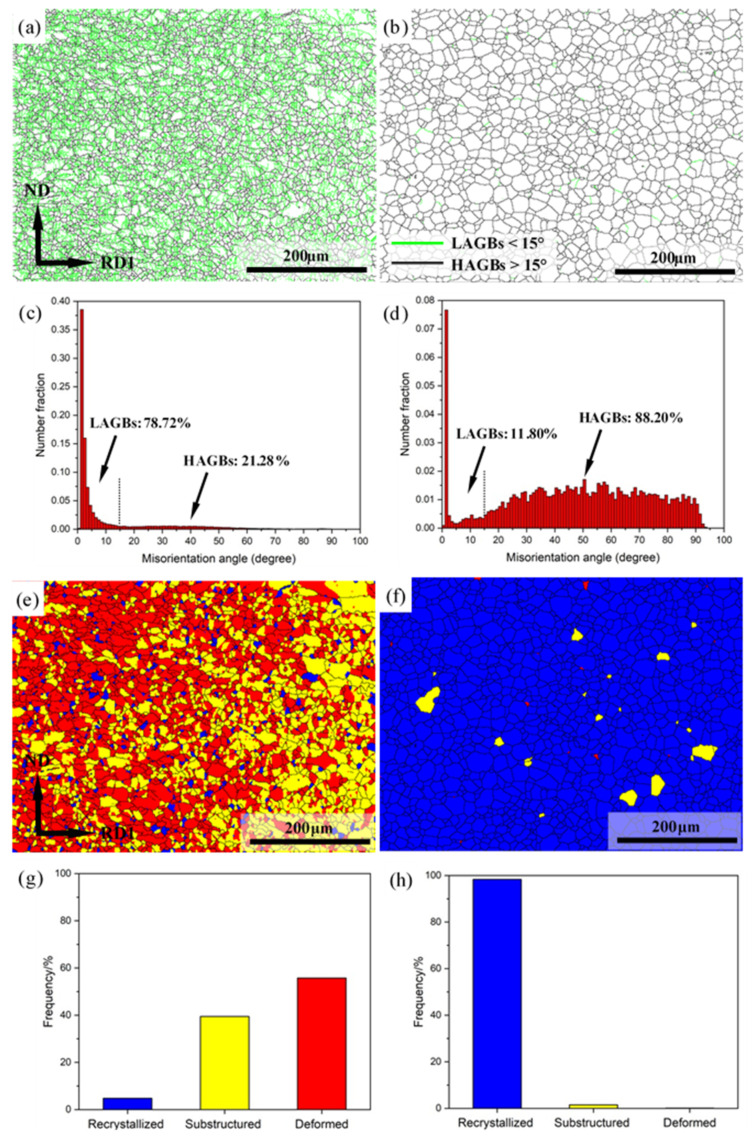
Grain boundary maps of (**a**) as-rolled and (**b**) as-annealed; misorientation angle distributions of (**c**) as-rolled and (**d**) as-annealed; structure component distribution maps of (**e**) as-rolled and (**f**) as-annealed; and structure fraction distributions of (**g**) as-rolled and (**h**) as-annealed Mg-3Y sheets.

**Figure 15:**
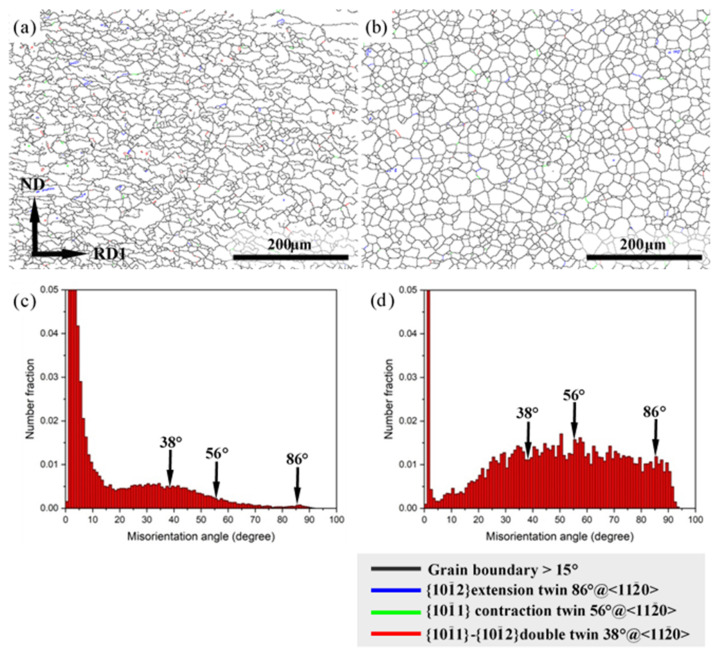
Misorientation angle maps showing various twin boundaries of (**a**) as-rolled and (**b**) as-annealed; misorientation angle distributions of (**c**) as-rolled and (**d**) as-annealed Mg-3Y sheets.

Grain boundary maps and misorientation angle distributions of Mg-3Y sheets are shown in Figure 14a–d. The high-angle grain boundaries (HAGBs > 15°) and low-angle grain boundaries (LAGBs < 15°) are characterized by black and green lines, respectively. Combined with the misorientation angle distributions, it can be seen that the proportion of LAGBs (78.72%) is considerably higher than HAGBs (21.28%) in Mg-3Y-R sheet. Indeed, the number fraction of boundaries with low misorientation angles (<10°), which are a result of lattice distortion and subgrain formation within grains caused by dislocation slip, is 75.12% and dominates the major proportion. Intersection of {10-12} extension twins can lead to the formation of twin boundaries with a low misorientation angle of ~7.4° [45]. However, a comparison of the twin boundary map (described below in Figure 15a) with the low-angle boundary map reveals that Mg-3Y-R sheet does not contain low-angle boundaries formed by this twin intersection. In contrast, the proportion of HAGBs (88.20%) is considerably higher than LAGBs (11.80%) in Mg-3Y-RA sheet. Based on [46], the proportion of HAGBs is related to the amount of recrystallized and deformed grains in the microstructure. A large amount of recrystallized grains would result in high proportion of HAGBs, meanwhile a large amount of deformed grains would result in high proportion of LAGBs. The recrystallization fraction of Mg-3Y-RA sheet is almost 100%, but the recrystallization fraction is only 4.79% for Mg-3Y-R sheet (Figure 14e–h). Relative complete SRX process produces a greater proportion of HAGBs. Furthermore, this results in more random grain orientation distribution and clear weakening of the (0002) texture.

Misorientation angle maps depicting various twin boundaries and corresponding misorientation angle distributions of Mg-3Y sheets are shown in Figure 15. The main twin types present in Mg-3Y sheets are {10-12} extension twin, {10-11} contraction twin, and {10-11}-{10-12} double twin. Despite the appearance of the different types of twins in both Mg-3Y-R and Mg-3Y-RA sheets, the fraction of twins is very small. The proportions of {10-12} extension twin, {10-11} contraction twin, and {10-11}-{10-12} double twin are 0.08%, 0.18%, and 0.47% for Mg-3Y-R sheet, respectively. The total proportion of twins is about 0.73%, less than 1.0%. As for Mg-3Y-RA sheet, the proportions of {10-12} extension twin, {10-11} contraction twin, and {10-11}-{10-12} double twin are 1.01%, 1.49%, and 1.11%, respectively. Although the total proportion (about 3.61%) of twins has some increase after annealing, the proportion is still very small. The increase in twins proportions may be attributed to the following two possible reasons. The reduction of total grain boundaries quantity results from the growth of grains and the preservation of twins during annealing. In addition, the preservation of twins indicates that the twins cannot act as the favorable nucleation site for SRX during subsequent short-term annealing. Combined with the analysis about the change of morphologies and number density of kink bands, the kink bands should be the favorable nucleation site for SRX except for grain boundaries. As mentioned above, few twins prove that the deformation bands should be kink bands, but twins again.

**Figure 16:**
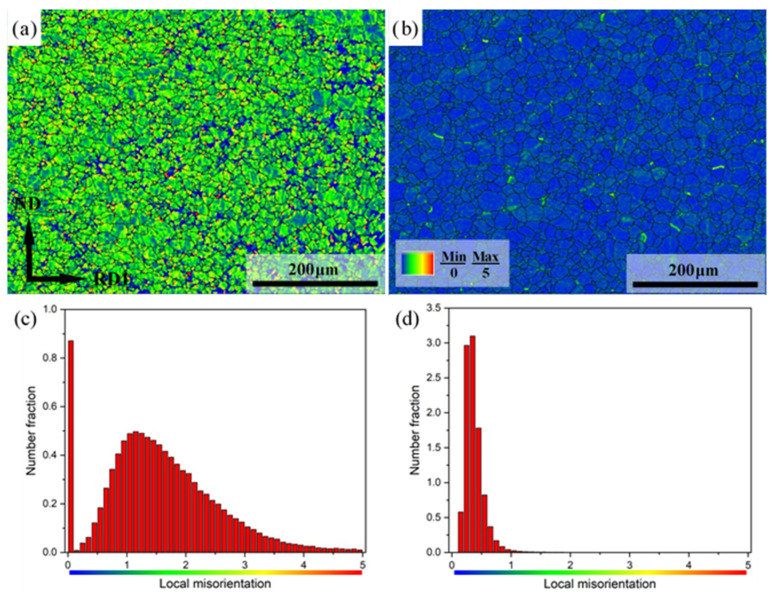
KAM maps of (**a**) as-rolled, (**b**) as-annealed, and corresponding local misorientation distributions of (**c**) as-rolled and (**d**) as-annealed Mg-3Y sheets.

The kernel average misorientation (KAM) maps and corresponding local misorientation distributions of Mg-3Y sheets are shown in Figure 16. The KAM map is constructed based on the average misorientation between a measuring point and all its neighbors. Consequently, the local misorientation and strain energy can be clearly reflected in the KAM map [47,48]. In the KAM maps, the KAM values indicate local misorientation, which we interpret in terms of density of geometrically necessary dislocations to provide the local strain value (high KAM value = high misorientation = high strain value). The high strain energy zone could be clearly observed by the red and orange colors. The relatively uniform distribution of similar colors indicates that the deformation of Mg-3Y-R sheet is comparatively homogenous except for a few high strain energy zones. The average KAM value of Mg-3Y-R sheet is found to be higher and drops from 1.57 to 0.38 remarkably after subsequent short-term annealing. Therefore, most of the areas nearly exhibit a strain-free state, indicating that SRX is a process of softening the work hardening effect [49,50]. The release of residual stress is beneficial to the improvement of ductility and stretch formability of Mg-3Y sheets.

**Figure 17:**
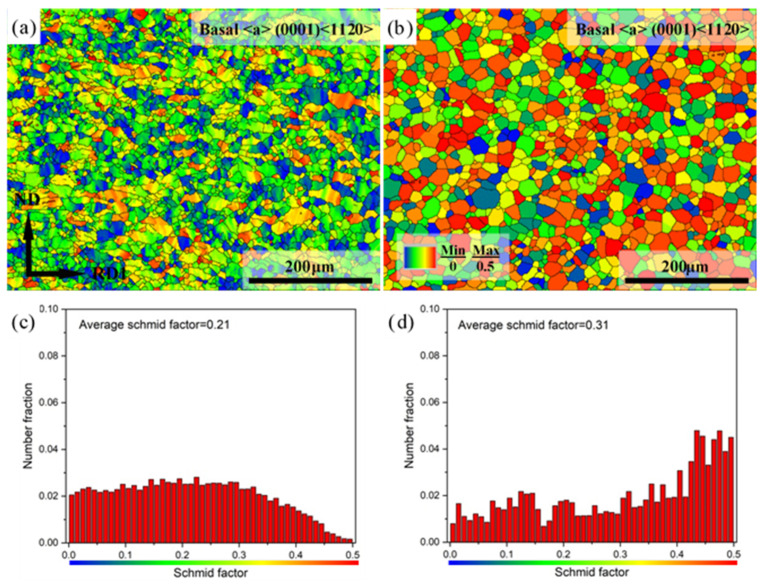
Basal <a> slip SF maps of (**a**) as-rolled, (**b**) as-annealed, and corresponding SF distributions of (**c**) as-rolled and (**d**) as-annealed Mg-3Y sheets.

To fully understand the effect of annealing on the ductility of Mg-3Y sheets, the Schmid factor (SF) distributions of the (0001)<11-20> basal <a> slip, (1-100)<11-20> prismatic <a> slip, (1-101)<11-20> pyramidal <a> slip, and (11-22)<11-2-3> pyramidal <c+a> slip is investigated by EBSD analysis. Figure 17 shows the (0001)<11-20> basal <a> slip SF maps and corresponding SF distributions of Mg-3Y sheets. The blue grains usually have their basal planes parallel to the tensile direction, i.e., along the RD2 direction. The blue grains have a relatively lower basal <a> slip SF and is unfavorable for basal <a> slip. However, the red grains usually have their basal planes inclined by about 45° to the tensile planes. Therefore, the red grains have a higher basal <a> slip SF, which is favorable for basal <a> slip. Compared with Mg-3Y-R sheet, a large number of red grains are observed in Mg-3Y-RA sheet, which is correlated with the weak texture resulting from SRX during annealing. As can be seen from the fraction distribution of SF for basal <a> slip of both sheets before and after annealing, the number fraction of grains gradually decreases with the increasing SF values (with regard to SF values variation range of 0.3–0.5) in Mg-3Y-R sheet. The number fraction of grains with the highest SF values (> 0.4) [51] corresponds to the lowest number fraction of 7.17%. In contrast, there is a gradual increase in SF distribution for Mg-3Y-RA sheet. The number fraction of grains with a high SF value (> 0.4) is greater than 38.24%. Correspondingly, the average SF for basal <a> slip increases from 0.21 to 0.31 after annealing. Therefore, the activation of (0001)<11-20> basal <a> slip is further enhanced in Mg-3Y-RA sheet.

**Figure 18:**
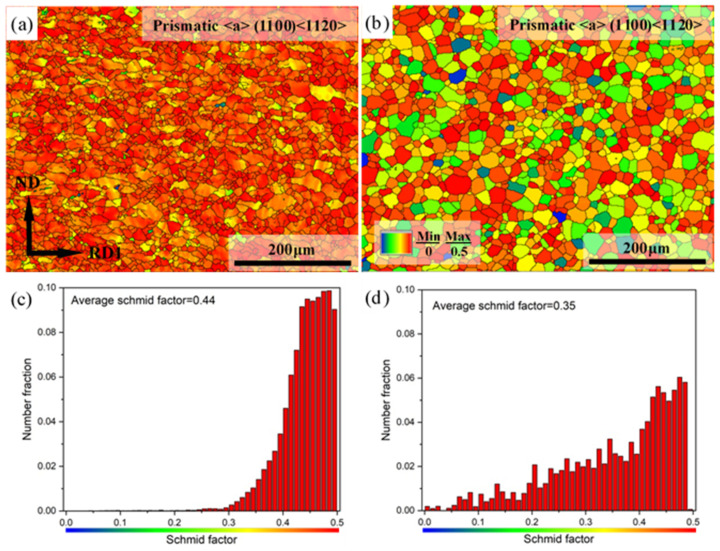
Prismatic <a> slip SF maps of (**a**) as-rolled, (**b**) as-annealed, and corresponding SF distributions of (**c**) as-rolled and (**d**) as-annealed Mg-3Y sheets.

The (1-100)<11-20> prismatic <a> slip SF maps and corresponding SF distributions of Mg-3Y sheets are shown in Figure 18. As mentioned above, the prismatic planes of blue grains are usually parallel to the tensile direction and have relatively lower prismatic <a> slip SF, which is unfavorable for prismatic <a> slip. The prismatic planes of red grains are usually inclined by about 45° to the tensile planes and have a higher prismatic <a> slip SF, which is favorable for prismatic <a> slip. The colors of all grains are approaching the red color, which indicates that prismatic <a> slip SF values are high for Mg-3Y-R sheet. The number fraction of grains with high prismatic <a> slip SF values (>0.4) is about 84.13% and almost all the SF values of grains are more than 0.3. Nevertheless, the number fraction of grains with high prismatic <a> slip SF values decreases to 46.15% for Mg-3Y-RA sheet. In addition, some grains show unfavorable orientation for prismatic <a> slip. Nevertheless, the average SF for prismatic <a> slip decreases from 0.44 to 0.35 after annealing. Moreover, the activation of (1-100)<11-20> prismatic <a> slip (smaller value of average SF: 0.35) is still higher than (0001)<11-20> basal <a> slip (larger value of average SF: 0.31) for both Mg-3Y sheets.

**Figure 19:**
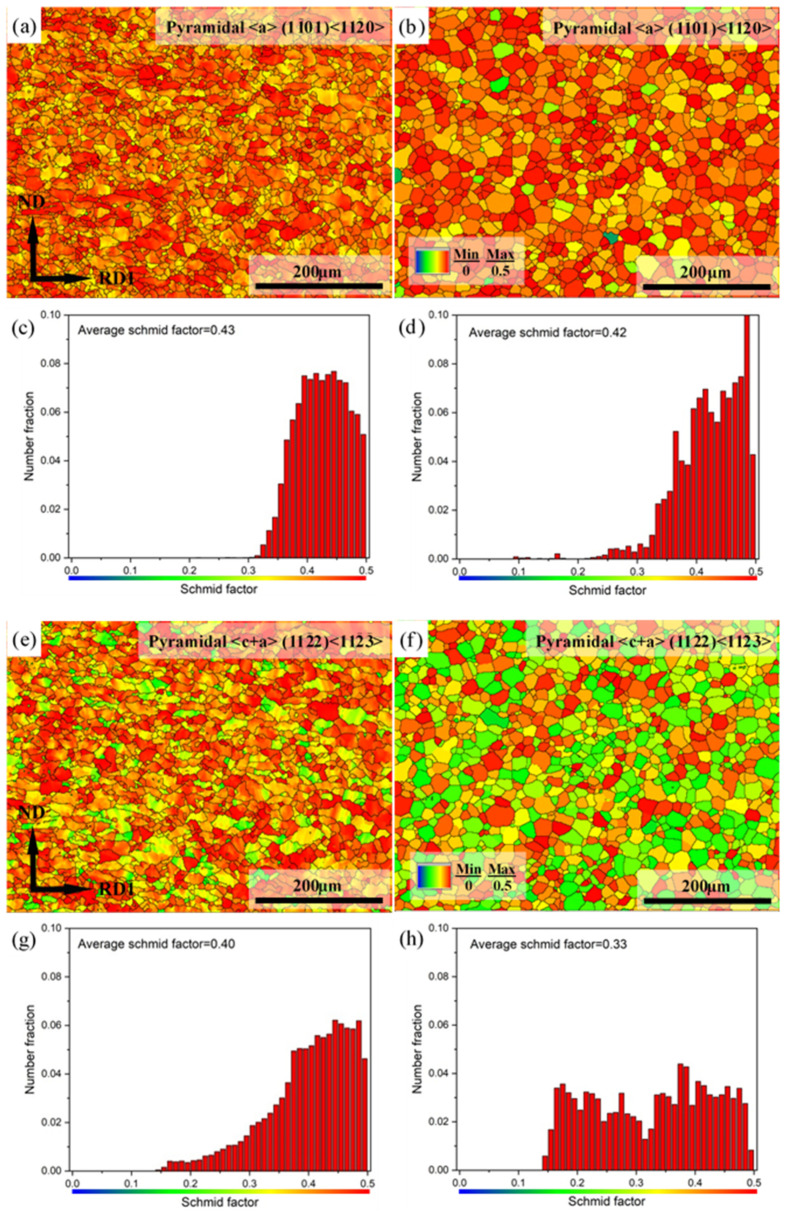
Pyramidal <a> slip SF maps of (**a**) as-rolled, (**b**) as-annealed, and corresponding SF distributions of (**c**) as-rolled and (**d**) as-annealed; pyramidal <c+a> slip SF maps of (**e**) as-rolled, (**f**) as-annealed, and corresponding SF distributions of (**g**) as-rolled and (**h**) as-annealed Mg-3Y sheets.

Figure 19 shows the pyramidal slip (including pyramidal <a> slip and pyramidal <c+a> slip) SF maps and corresponding SF distributions of Mg-3Y sheets. Similarly, blue grains with relatively lower pyramidal slip SF are unfavorable for pyramidal slip. The red grains with a higher pyramidal slip SF are favorable for pyramidal slip. The (1-101)<11-20> pyramidal <a> slip SF maps and corresponding SF distributions are displayed in Figure 19a–d. The number fractions of grains with high pyramidal <a> slip SF values (>0.4) are about 69.05% and 68.33% for Mg-3Y-R and Mg-3Y-RA sheets, respectively. Nearly all the SF values of grains are more than 0.3 for both Mg-3Y-R and Mg-3Y-RA sheets. The average SF values for pyramidal <a> slip have no clear change and maintain the same level before and after annealing. Therefore, annealing has little effect on the activation of (1-101)<11-20> pyramidal <a> slip. The (11-22)<11-2-3> pyramidal <c+a> slip SF maps and corresponding SF distributions are displayed in Figure 19e–f. The number fractions of grains with high pyramidal <c+a> slip SF values (>0.4) are about 56.75% for Mg-3Y-R sheets. After annealing, the number fraction of grains with high pyramidal <c+a> slip SF values drops to 29.48% for Mg-3Y-RA sheet. Accordingly, the average SF for pyramidal <c+a> slip decreases from 0.40 to 0.33 after annealing. Similar to (1-100)<11-20> prismatic <a> slip, the activation of (11-22)<11-2-3> pyramidal <c+a> slip (smaller value of average SF: 0.33) is higher than (0001)<11-20> basal <a> slip (larger value of average SF: 0.31) for both Mg-3Y sheets.

Combined with the analysis of SF above, it can be concluded that the addition of Y into Mg promotes the activity of non-basal slip [52,53]. Both average SF values of prismatic <a> slip, pyramidal <a> slip, and pyramidal <c+a> slip are greater than 0.30 for Mg-3Y-R and Mg-3Y-RA sheets, which show a high activation (Table 2). The Mg-3Y-RA sheet has a crystallographic orientation, which is more favorable for basal <a> slip, but a little unfavorable for prismatic <a> slip and pyramidal <c+a> slip after SRX. Therefore, the average SF values of basal <a> slip increase, but decrease for prismatic <a> slip and pyramidal <c+a> slip. The enhanced activity of basal <a> slip could be beneficial for the improvement of ductility and stretch formability at room temperature for Mg-3Y-RA sheet.

### 3.3. Macro-Texture

**Figure 20:**
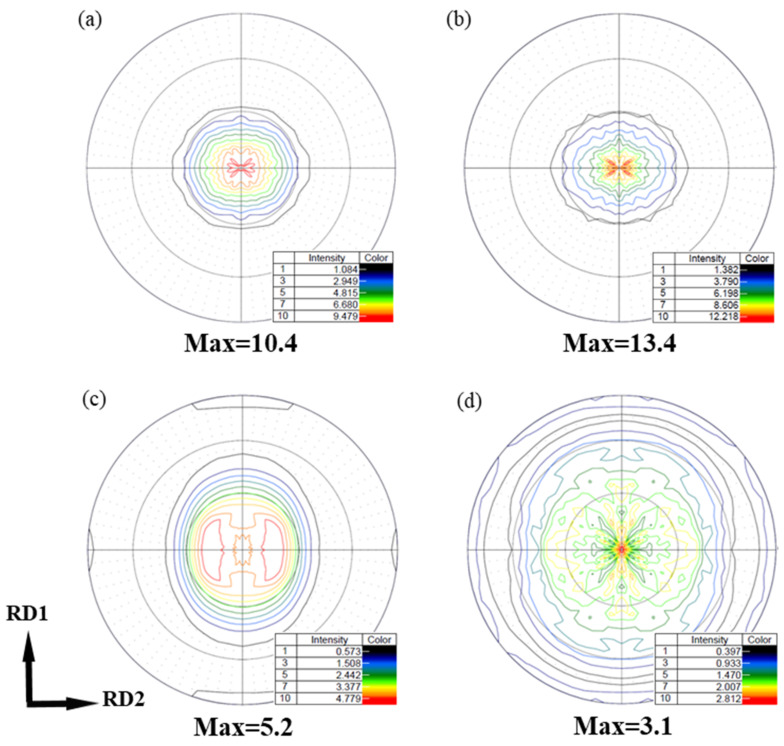
X-ray recalculated (0002) pole figure of (**a**) as-rolled and (**b**) as-annealed pure Mg sheets; (**c**) as-rolled and (**d**) as-annealed Mg-3Y sheets.

The macro-texture of the sheets by means of the (0002) pole figures are displayed in Figure 20. As expected, a typical strong basal texture is formed in Mg-R sheet, where the orientation distribution of most basal poles is parallel to the normal direction (Figure 20a). After annealing, the texture intensity increases from 10.4 to 13.4 MRD, owing to the coarsening of grain in Mg-RA sheet, as shown in Figure 20b.

**Figure 21:**
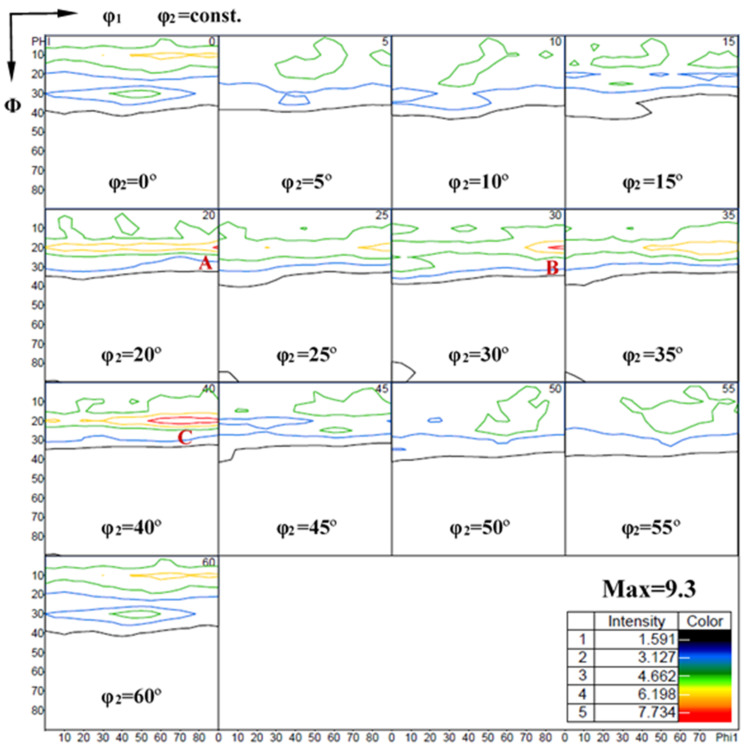
ODF sections of as-rolled Mg-3Y sheet (A, B and C are different texture component mark).

**Figure 22:**
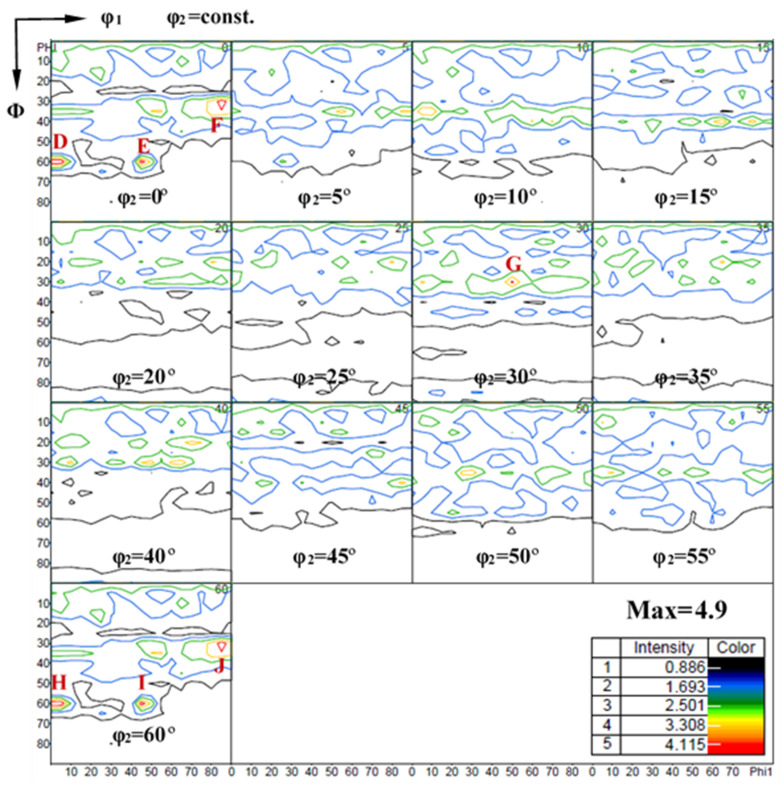
ODF sections of as-annealed Mg-3Y sheet (D, E, F, G, H, I and J are different texture component mark).

In contrast, the qualitative character of the Mg-3Y-R sheet texture is clearly distinct from typical basal texture. The texture has a clearly broader distribution of basal poles, as compared with its distribution in the Mg-R sheet. It was reported that the basal pole for many Mg alloys containing RE elements would be simple to spread from ND toward transverse direction (TD), resulting in an ellipse-shape orientation distribution of the (0002) basal texture [11,54]. However, in our cross-rolling case, the RD1 and RD2 in turn serve as the TD in a conventional unidirectional rolling. The basal pole would spread toward RD2 and RD1 by turning. Therefore, the (0002) basal texture of Mg-3Y-R sheet shows a subrotund orientation distribution (Figure 20c), which is beneficial to reduce the planar anisotropy between TD and RD [55]. Furthermore, the Mg-3Y-R sheet shows a split of the basal-pole intensity peak from ND toward RD2, which results in the formation of a double peak texture. This RD-splitting characteristic may be considered as the result of activation of pyramidal <c+a> slip [7,56]. Compared with Mg-R sheet, the maximum intensity of Mg-3Y-R sheet decreases from 10.4 to 5.2 MRD and the new texture components are formed. To reveal the change of texture and components, the orientation distribution function (ODF) sections of Mg-3Y-R sheet are presented in Figure 21. The major texture components of Mg-3Y alloy sheets are summarized in Table 3. There are three relatively stronger texture fibers with some dominant components in Mg-3Y-R sheet. The first consists of main texture component of A-{01-17}<-1-231> orientation grains, the second consists of main texture component of B-{0001}<-1-231> orientation grains, and the third consists of main texture component of C-{10-17}<-1-231> orientation grains. After annealing, a considerably weaker sheet texture is obtained in Mg-3Y-RA sheet compared with the Mg-3Y-R sheet. The orientation distribution of basal poles becomes more randomized and the (0002) basal maximum intensity further reduces to 3.1 MRD. The tendency of macro-texture intensity variety is the same as the micro-texture mentioned above. The double peak texture disappears and a new multiple-peak texture appears. High deformation temperature enhances the activities of non-basal slips and GBS, which may affect the SRX kinetics. Deformation at higher temperatures increases the orientation gradients near grain boundaries due to GBS and/or shearing connected with grain boundary serration [57]. In the previous work, some of the authors reported that a significant weakening of texture may be achieved by annealing a rolled AZ31 alloy with a deformation microstructure without an occurrence of DRX [23,24], which has also been revealed to be due to discontinuous SRX at pre-existing grain boundaries, i.e., the large orientation gradients and the high local dislocation densities near grain boundaries are likely to induce SRX at pre-existing grain boundaries [58]. Therefore, it can be expected that the textures of the sheet rolled at higher temperatures weaken more remarkably during annealing if plenty of pre-existing grain boundaries are contained. Consequently, strong SRX induced by plenty of pre-existing grain boundaries should be the reason for the change of texture character in Mg-3Y-RA sheet. In the same way, the ODFs of Mg-3Y-RA sheet are presented in Figure 22 to reveal the change of texture and components after annealing. The texture fibers and components vanish and numerous weakened discrete textures and components emerge. Not only the scatter is increased, but also the intensity is significantly decreased. There are seven relatively stronger discrete textures with different dominant components (D-J orientation grains) in Mg-3Y-RA sheet. As pointed by Suh B C et al. [59,60], this multiple-peak texture is beneficial in obtaining good formability since a large number of grains are favorably oriented for basal <a> slip to operate during stretch forming in comparison with the RD-split texture developed in the AXM100 alloy.

### 3.4. Mechanical Properties

**Figure 23:**
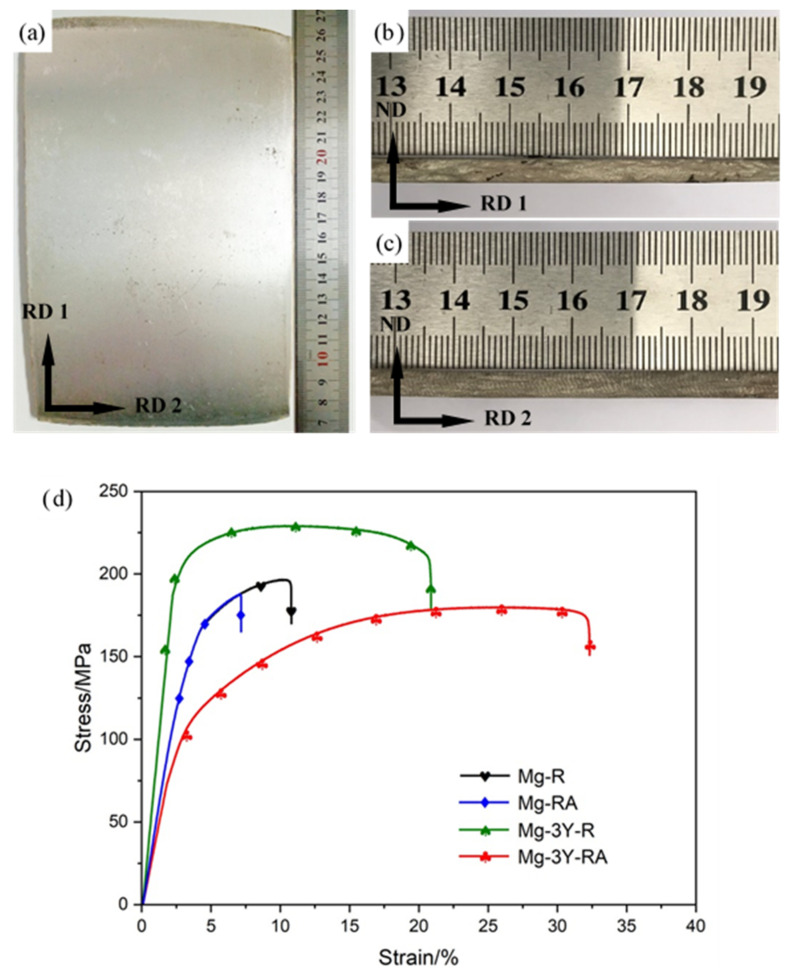
Macro-morphologies of as-rolled Mg-3Y alloy sheet obtained from (**a**) RD1-RD2 plane, (**b**) RD1-ND plane, (**c**) RD2-ND plane, and (**d**) typical engineering stress-strain curves of tensile test for all sheets at room temperature.

The macro-morphologies of the final as-rolled Mg-3Y alloy sheet are shown in Figure 23a–c. No crack exists in Mg-3Y-R sheet, indicating the ideal rollability. The tensile stress-strain curves of the sheets at room temperature are shown in Figure 23d. The 0.2% yield strength (YS), ultimate tensile strength (UTS), and elongation to failure (FE) are summarized in Table 4. The YS, UTS, and FE of the Mg-R sheet are 142 ± 5 MPa, 196 ± 7 MPa, and 6.4 ± 0.3%, respectively. In contrast, the room temperature YS, UTS, and FE of Mg-RA sheet slightly decrease to 140 ± 3 MPa, 187 ± 5 MPa, and 4.6 ± 0.4%, respectively, indicating worsening strength and ductility. The deterioration of mechanical properties should be attributed to the coarsening of grain during SRX.

Compared with Mg-R sheet, the YS and UTS of Mg-3Y-R sheet increase by about 60 and 32 MPa, respectively. The synergistic effect of grain refinement strengthening, second phase strengthening, solid solution strengthening, and dislocation strengthening improves the UTS. The improvement of YS could be contributed to the fine grain strengthening, in accordance with the Hall-Petch relation [61]. As for ductility, the FE of Mg-3Y-R sheet is about three times the size of Mg-R sheet. The weakened double peak texture and the refined grains should be the main reason for the improvement of ductility. As the tensile flow curve shows, the Mg-3Y-RA sheet exhibits a gradual work hardening behavior and a prolonged portion of plastic instability after necking in the tensile curves. Although the YS and UTS of Mg-3Y-RA sheets are decreased to 108 ± 6 MPa and 180 ± 8 MPa, respectively compared with Mg-3Y-R sheet due to the weakening effect of grain refinement strengthening and dislocation strengthening, the FE further increases to 25.6 ± 0.8% after annealing. High ductility of Mg-3Y-RA sheet should be derived from the following four aspects: (1) Significantly reduced kink bands. In tensile process, these existing kink bands would obstruct the subsequent dislocation motion, and easily act as initiation sites for crack nucleation and propagation. This would result in the deterioration of the ductility. (2) Enhanced activity of (0001)<11-20> basal <a> slip. Basal <a> slip could be more easily activated at lower critical resolved shear stress due to the increased average SF values of basal <a> slip. (3) Increased strain hardening behavior. It has been generally accepted that increasing the strain hardening could inhibit the onset of localized deformation, improve the uniform elongation, and therefore increase the elongation to failure [62,63]. (4) Texture weakening. The weakened texture can not only contribute to accommodating the basal slip in tension, but also have a significant impact on the twinning response [62].

**Figure 24:**
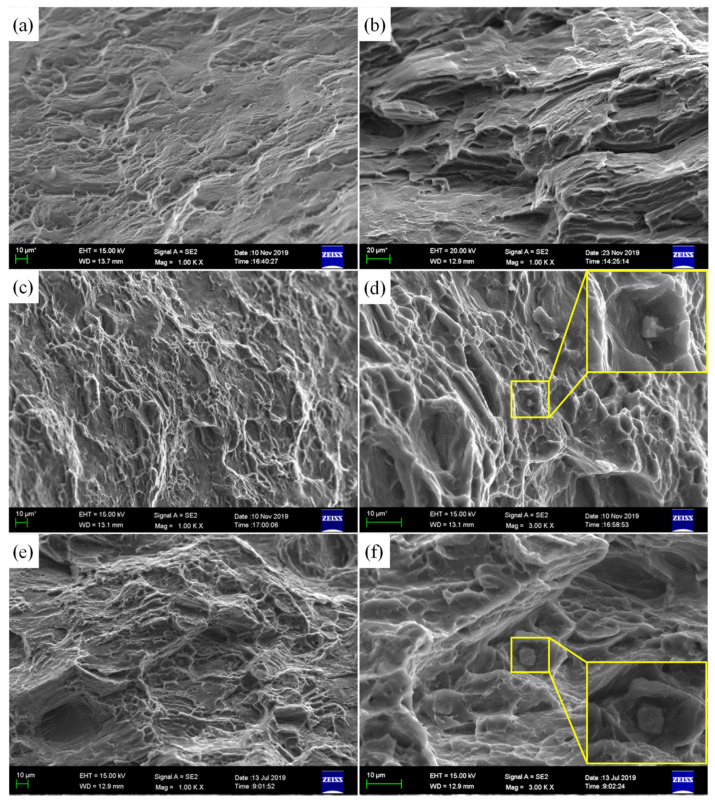
SEM micrographs of the fracture surface of (**a**) as-rolled and (**b**) as-annealed pure Mg sheets; (**c**,**d**) as-rolled and (**e**,**f**) as-annealed Mg-3Y sheets.

The SEM images of tensile fracture surface are presented in Figure 24. Both the Mg-R and Mg-RA sheets exhibit large cleavage facets, which contain tear ridges and a small number of dimples (Figure 24a,b). Therefore, this fracture mode can be categorized as a quasi-cleavage fracture [51]. However, in the Mg-3Y-R and Mg-3Y-RA sheets, a ductile fracture surface with elongated dimples is observed (Figure 24c,e). The cleavage facets are reduced and a tearing characteristic appears in Mg-3Y-RA sheet after annealing. The appearance of tear ridges should be originated from the grain growth during SRX. In addition, the cracked particles inside the dimples (marked by yellow rectangle squares in Figure 24d,f) are observed in both Mg-3Y-R and Mg-3Y-RA sheets. However, the Mg-3Y-RA sheet possesses deeper dimples compared with the as-rolled condition, indicating the highest room temperature ductility among all of the sheets. The change of fracture morphology corresponds well with the improvement of elongation.

### 3.5. Stretch Formability

**Figure 25:**
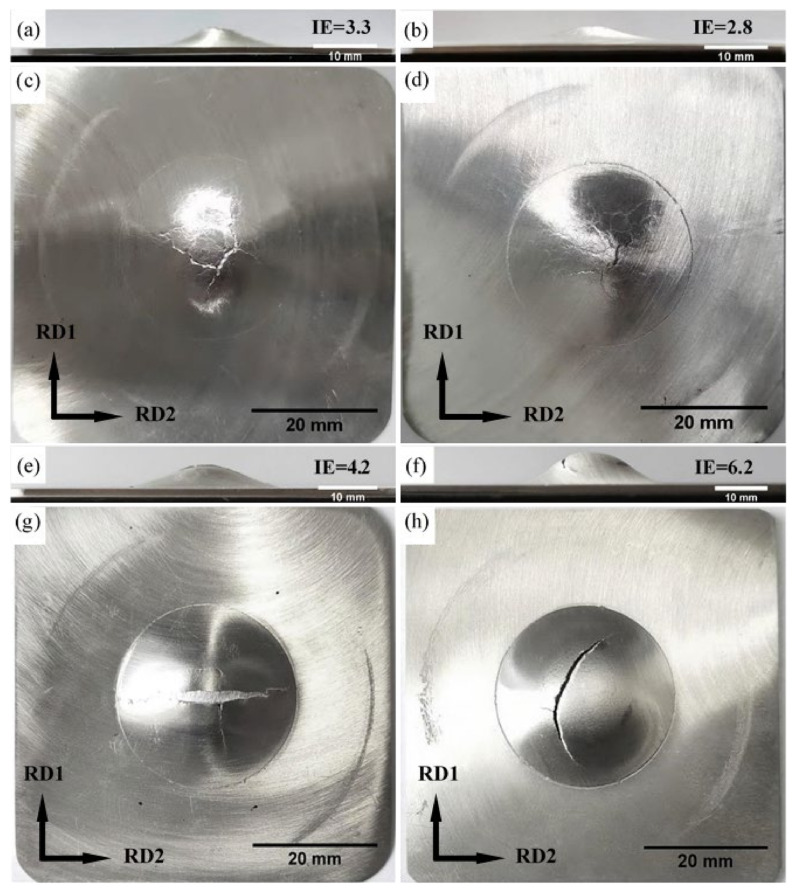
Macro-morphology of specimens after the Erichsen test at room temperature of (**a**,**c**) as-rolled and (**b**,**d**) as-annealed pure Mg sheets; (**e**,**g**) as-rolled and (**f**,**h**) as-annealed Mg-3Y sheets.

The results of Erichsen test are summarized in Table 5. Both Mg-R and Mg-RA sheets show a poor stretch formability, especially Mg-RA sheet. The index Erichsen (IE) value of Mg-3Y-R sheet is 4.18 ± 0.08 mm, which exhibits relatively good formability compared with Mg-R and Mg-RA sheets. The improvement of formability originated from the reduction of basal texture intensity (from 10.4 to 5.2 MRD) and inclination of basal pole (about ±20° tilted to RD2) [15,64,65,66]. The IE value of Mg-3Y-RA sheet is significantly increased from 4.18 ± 0.08 mm to 6.22 ± 0.05 mm after annealing. The Mg-3Y-RA sheet performs a more significant stretch formability, which is attributed to the further weakened texture. Macro-morphology of pure Mg and Mg-3Y sheets after the Erichsen test are shown in Figure 25. Top views of the pure Mg sheets after the Erichsen test reveal that both Mg-R and Mg-RA sheets exhibit a surface crack splitting along multiple directions, which corresponds to the strong butterfly-shaped basal texture (Figure 25c,d). However, the top view of the Mg-3Y-R sheet, which has a basal texture with a splitting of basal pole toward the RD2, exhibits a surface crack parallel to the RD2 after the Erichsen test (Figure 25g). In addition, the circular arc shaped surface crack appears along the angle between RD1 and RD2 in Mg-3Y-RA sheet, which could be related to the randomization of texture (Figure 25h). These results indicate that the splitting of basal pole toward the RD2 and the change of the texture character would strongly affect the macroscopic fracture behaviors of the sheets during Erichsen tests.

## 4. Conclusions

In this study, a multi-pass high temperature cross-rolling with inter-pass annealing was applied to Mg-3Y alloy. With pure Mg as a reference, the microstructures, texture, mechanical properties, and stretch formability of Mg-3Y alloy were investigated. The main conclusions are summarized as follows:

(1) The morphological characteristic of β-Mg_24_Y_5_ phases with micro-nano size coexistence is formed in Mg matrix, which should be relevant to the solidification segregation behavior of Y solute.
(2) The Mg-3Y-R sheet exhibits a relatively homogeneous deformed microstructure consisting of deformed grains with extensive kink bands and dispersed second phase particles. A double peak texture character appears in Mg-3Y-R sheet with a remarkably reduced pole density and a split of the texture peaks by about ±20° tilted to rolling direction 2.
(3) The Mg-3Y-RA sheet presents a complete SRXed microstructure consisting of uniform equiaxed grains. The double texture disappears and a weakened multiple-peak texture appears. The maximum pole density of (0002) basal plane is further decreased from 5.2 to 3.1 MRD. The change of texture that occurs in the Mg-3Y-RA sheet should be due to the strong SRX induced by kink bands and grain boundaries.
(4) Compared with the pure Mg, the Mg-3Y alloy sheet achieved a simultaneous improvement of ductility and stretch formability via high temperature cross-rolling and subsequent short-term annealing. High ductility and stretch formability are attributed to the fine dispersed β-Mg_24_Y_5_ phases, homogeneous SRXed microstructure, enhanced activity of basal <a> slip and non-basal slip, and weakening of texture.

## Figures and Tables

**Table 1:** Chemical compositions of the as-cast materials (wt%).

Materials	Y	Mg
Mg	-	Bal.
Mg-3Y	2.90	Bal.

**Table 2:** Average Schmid factor (SF) of slip systems in Mg-3Y sheets.

Sheets	Average Schmid Factor (SF)			
	Basal <a> (0001)<11-20>	Prismatic <a> (1-100)<11-20>	Pyramidal <a> (1-101)<11-20>	Pyramidal <c+a> (11-22)<11-2-3>
Mg-3Y-R	0.21	0.44	0.43	0.40
Mg-3Y-RA	0.31	0.35	0.42	0.33

**Table 3:** Major texture components of Mg-3Y alloy sheets.

Mg-3Y-R Sheet		Mg-3Y-RA Sheet	
Symbol	Texture Components	Symbol	Texture Components
		D	{01-11}<2-1-10>
		E	{01-11}<8-7-16>
A	{01-17}<-1-231>	F	{01-13}<0-332>
B	{0001}<-1-231>	G	{11-26}<1-542>
C	{10-17}<-1-231>	H	{10-11}<1-210>
		I	{10-11}<0-111>
		J	{10-13}<-4-153>

**Table 4:** Tensile properties of the sheets at room temperature.

Sheets	YS (MPa)	UTS (MPa)	FE (%)
Mg-R	142 ± 5	196 ± 7	6.4 ± 0.3
Mg-RA	140 ± 3	187 ± 5	4.6 ± 0.4
Mg-3Y-R	202 ± 4	228 ± 6	18.6 ± 0.6
Mg-3Y-RA	108 ± 6	180 ± 8	25.6 ± 0.8

**Table 5:** Stretch formability of the sheets at room temperature.

Sheets	Punch Force (kN)	IE (mm)
Mg-R	1.33 ± 0.03	3.3 ± 0.04
Mg-RA	1.19 ± 0.02	2.8 ± 0.06
Mg-3Y-R	2.02 ± 0.03	4.2 ± 0.08
Mg-3Y-RA	4.61 ± 0.04	6.2 ± 0.05

## Data Availability

The data presented in this study are available upon request from the corresponding author. The data are not publicly available due to the requirements of related projects.
